# Biodegradable, Water‐Resistant, Anti‐Fizzing, Polyester Nanocellulose Composite Paper Straws

**DOI:** 10.1002/advs.202205554

**Published:** 2022-11-20

**Authors:** Hojung Kwak, Hyeri Kim, Seul‐A Park, Minkyung Lee, Min Jang, Sung Bae Park, Sung Yeon Hwang, Hyo Jeong Kim, Hyeonyeol Jeon, Jun Mo Koo, Jeyoung Park, Dongyeop X. Oh

**Affiliations:** ^1^ Research Center for Bio‐based Chemistry Korea Research Institute of Chemical Technology (KRICT) Ulsan 44429 Republic of Korea; ^2^ Department of Plant and Environmental New Resources Kyung Hee University Yongin 17104 Republic of Korea; ^3^ Department of Organic Materials Engineering Chungnam National University Daejeon 34134 Republic of Korea; ^4^ Department of Chemical and Biomolecular Engineering Sogang University Seoul 04107 Republic of Korea

**Keywords:** paper straws, cellulose nanocrystals, water resistance, marine biodegradation, anti‐fizzing

## Abstract

Among plastic items, single‐use straws are particularly detrimental to marine ecosystems because such straws, including those made of poly(lactic acid) (PLA), are sharp and extremely slowly degradable in the ocean. While paper straws are promising alternatives, they exhibit hydration‐induced swelling even when coated with a non‐degradable plastic coating and promote effervescence (fizzing) in soft drinks owing to their surface heterogeneities. In this study, upgraded paper straw is coated with poly(butylene succinate) cellulose nanocrystal (PBS/CNC) composites. CNC increases adhesion to paper owing to their similar chemical structures, optimizes crystalline PBS spherulites through effective nucleation, and reinforces the matrix through its anisotropic and rigid features. The straws are not only anti‐fizzing when used with soft drinks owing to their homogeneous and seamless surface coatings, but also highly water‐resistant and tough owing to their watertight surfaces. All degradable components effectively decompose under aerobic composting and in the marine environment. This technology contributes to United Nations Sustainable Development Goal 14 (Life Below Water).

## Graphical abstract

Hybrid bioplastic paper straws are successfully fabricated with poly(butylene succinate) (PBS) and PBS oligomer modified cellulose nanocrystals (BS‐CNC). PBS/BS‐CNC paper straw has highly robust mechanical and physical properties, user‐friendly properties by acting as an anti‐fizzing effect, and outstanding biodegradability under aerobic and marine conditions. PBS/BS‐CNC straws exhibit the considerable potential of bioplastics' practical applications.

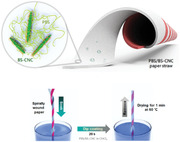

## 1 Introduction

**Figure 1:**
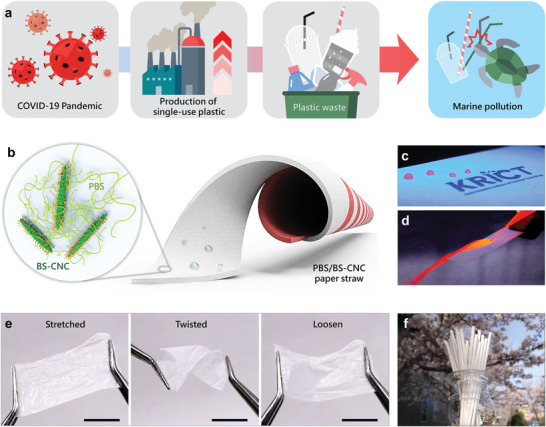
a) Overview depicting increasing plastic waste in the marine environment during the COVID‐19 pandemic. b) Schematic illustration of a PBS/BS‐CNC paper straw. Images showing c) a droplet and d) flowing water on PBS/BS‐CNC paper surfaces. e) The PBS/BS‐CNC coating film is able to recover its original shape after being stretched and twisted. Scale bar: 1 cm. f) Photographic image showing PBS/BS‐CNC paper straws.

Plastics are generally lightweight, chemically stable, flexible, and tunable; consequently, they are essential in everyday life because they can be used in countless applications.^[^ ^1^, ^2^, ^3^ ^]^ For sanitation reasons, the past few years have witnessed the explosive consumption of plastic products in applications such as masks and disposable food‐related plastics during the SARS‐CoV‐2 coronavirus (COVID‐19) pandemic, in which an estimated 530 Mt of plastic waste was produced annually (**Figure** **1**a).^[^ ^4^, ^5^, ^6^, ^7^ ^]^

Among plastic items, single‐use straws are particularly detrimental to the marine environment^[^ ^8^, ^9^ ^]^ because they are sharp and can mechanically injure sea animals.^[^ ^10^ ^]^ In addition, they are rarely recycled because they are small, which makes collection difficult; they are also non‐degradable.^[^ ^11^, ^12^, ^13^ ^]^ While poly(lactic acid) (PLA) is a biodegradable plastic, straws made from PLA are not marine‐degradable, which we confirmed in a marine experiment (vide infra).

Plastic‐coated paper straws are emerging globally as sustainable alternatives to non‐degradable straws.^[^ ^14^, ^15^ ^]^ Unfortunately, cellulosic paper and hydrophobic plastic are poorly compatible, which leads to the formation of uneven coatings with sections of uncovered paper.^[^ ^16^, ^17^ ^]^ As a result, paper straws absorb moisture and are easily torn^[^ ^18^ ^]^; they also promote effervescence (fizzing) in soft drinks (i.e., carbonated drinks) because their heterogeneous surfaces act as nucleation sites that condense supersaturated carbon dioxide.^[^ ^19^ ^]^ Overflowing fizzing drinks and a damp straw texture lead to poor customer satisfaction. Most importantly, the non‐degradable plastics used to coat materials generate microplastics through weathering,^[^ ^20^, ^21^, ^22^, ^23^ ^]^ Alternatively, while biodegradable polyesters have also been introduced as paper straw coatings, they do not effectively prevent swelling or fizzing due to their poor physical properties. For example, poly(butylene succinate) (PBS), as a representative biodegradable plastic, adheres poorly to paper owing to its relatively high hydrophobicity.^[^ ^24^ ^]^

Cellulose is the most abundant natural polymer on Earth and is also the main component of paper.^[^ ^25^, ^26^, ^27^, ^28^ ^]^ Native cellulose is a semicrystalline one‐dimensional (1D) fibril material consisting of crystalline and amorphous regions with high aspect ratios.^[^ ^29^, ^30^, ^31^ ^]^ Crystalline cellulose fibrils are very strong (up to 7.6 GPa)^[^ ^32^ ^]^ and stiff (up to 160 GPa)^[^ ^33^ ^]^ because they are stabilized through intra‐ and intermolecular hydrogen bonds,^[^ ^34^, ^35^ ^]^ van der Waals forces,^[^ ^36^ ^]^ and hydrophobic interactions.^[^ ^37^ ^]^ Cellulose crystals are crystalline nanomaterials; their extraction from cellulose biomass by acid treatment advantageously gives cellulose nanocrystals (CNC),^[^ ^38^ ^]^ which are currently in the spotlight as sustainable filler materials because they are bio‐renewable and biodegradable. We hypothesized that effective PBS/CNC hybridization will provide excellent adhesion to paper substrates and improve water resistance since CNC is very similar in nature to paper and is an effective reinforcing filler.^[^ ^39^, ^40^, ^41^ ^]^

In this study, we prepared a new class of paper straw coated with a PBS‐CNC composite to endow it with anti‐fizzing properties, high water resistance, mechanical robustness, and high degradability under aerobic conditions and in the marine environment. The CNC surface was modified by a PBS oligomer (BS‐CNC) to enable uniform dispersion in a hydrophobic PBS matrix (Figure 1b). A paper straw was fabricated by spirally winding two pieces of filter paper and dipping it into a PBS/BS‐CNC suspension (Figure 1f). The surface of the paper straw completely resists dripped and flowing water (Figure 1c,d). BS‐CNC endowed the composite coating film with improved mechanical properties. The film coating is flexible and can be plied into desired shapes that can be stretched, twisted, and loosened according to the required purpose (Figure 1e). The novel straws do not promote soft‐drink fizzing or severe effervescence in carbonated beverages because the well‐dispersed PBS/BS‐CNC coating provides a homogenous and seamless surface that is ascribable to good CNC/paper affinity. Most importantly, they are fully degraded during aerobic composting as well as in real marine environments, owing to the use of biodegradable materials. PBS/BS‐CNC paper straws overcome the disadvantages associated with traditional plastic‐ and paper‐straw‐based plastic waste.

## 2 Results and Discussion

**Figure 2:**
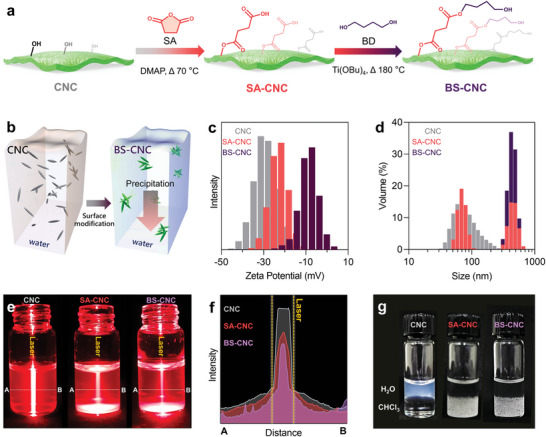
a) Modifying the CNC surface with succinic anhydride (SA) and 1,4‐butanediol (BD). b) Schematic image showing BS‐CNC aggregation in aqueous solution. c) Zeta potential and d) size distributions of CNC, SA‐CNC, and BS‐CNC in water. e) The Tyndall effect is observed when the centers of CNC, SA‐CNC, and BS‐CNC aqueous solutions are irradiated with a laser. f) Light intensities as functions of A–B distance in panel. g) Dispersions of CNC, SA‐CNC, and BS‐CNC in water/chloroform mixtures.

CNC easily aggregates in the PBS matrix owing to differences in hydrophilicity and polarity, which limits the nucleating and reinforcing effects of CNC. To overcome this limitation, the CNC surface was modified with PBS oligomers in a two‐step esterification process (**Figure** **2**a). The hydroxyl groups of CNC were first modified with succinic anhydride (SA) using ring‐opening chemistry (to give SA‐CNC), after which it was continuously esterified with 1,4‐butanediol (BD) at 180 °C to give PBS‐oligomer‐modified CNC surfaces (i.e., BS‐CNC).

The chemical structures of CNC, SA‐CNC, and BS‐CNC were examined by ^13^C nuclear magnetic resonance (NMR) spectroscopy and Fourier‐transform infrared (FT‐IR) spectroscopy (Figure S1a‐c, Supporting Information). BS‐CNC exhibited the most intense methylene peak by ^13^C‐NMR spectroscopy, which indicates that the CNC surface had been sequentially modified with SA and BD in the two‐step esterification process. The peak at 1720 cm^−1^ in the FT‐IR spectrum of SA‐CNC is assigned to carbonyl‐group stretching; this peak is broader in the spectrum of BS‐CNC, which is ascribable to the second esterification step involving BD. In addition, the intensity of the hydroxyl peak at 3330 cm^−1^ was observed to decrease as the two‐step reaction progressed.

The degree of esterification of each modified CNC sample was evaluated based on its aqueous dispersion. Both BS‐ and SA‐CNC gradually precipitated in aqueous solution, unlike CNC (Figure 2b). The colloidal states of the various samples were further examined by measuring their zeta potentials and colloidal sizes in aqueous solution with a dynamic light scattering (DLS) instrument (Figure 2c,d). The magnitude of the zeta potential decreased with increasing degree of esterification, with values observed to change from −31.3 to −9.3 mV. The average size of the colloidal particles was observed to increase from 72.5 to 448.2 nm with increasing degree of esterification. Pristine CNC showed a much clearer Tyndall effect in aqueous solution than both modified CNCs (Figure 2e,f). On the other hand, both modified CNCs dispersed well in chloroform compared to the pristine CNC (Figure 2g).

**Figure 3:**
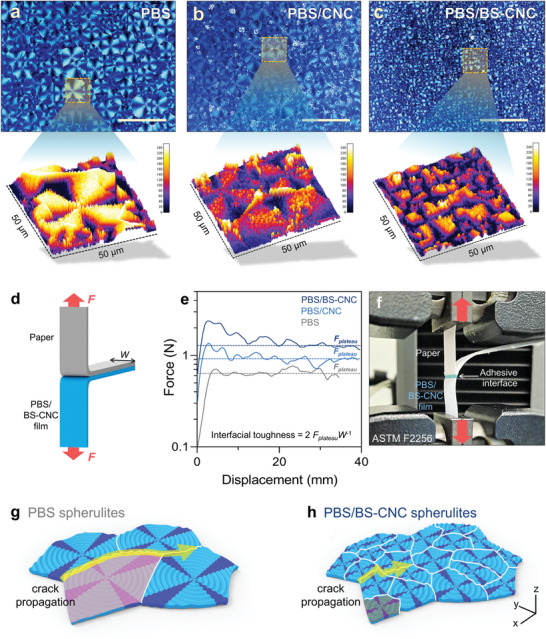
POM images of a) PBS, b) PBS/CNC, and c) PBS/BS‐CNC, with enlarged interactive ImageJ 3D surface plots below (yellow box, 50 × 50 µm). Scale bar: 100 µm. d) Schematic image showing the T‐peel test setup with PBS/BS‐CNC film and paper. e) PBS‐film/paper force‐displacement traces. *F*_plateau_ refers to the plateau region observed during T‐peel testing (ASTM F2256). f) Photographic image of the T‐peel test setup. g) Schematic image depicting crack propagation between PBS spherulites. h) Schematic image depicting crack propagation between PBS/BS‐CNC spherulites.

The structures of the crystalline spherulites in the PBS matrix are affected by the homogeneously dispersed BS‐CNC; the size and number of spherulites are controlled by CNC as the nucleating agent, with resulting changes affecting the mechanical properties of the PBS composite. In addition to pristine PBS, we prepared two different composites using 1 wt.% CNC and BS‐CNC. Polarized optical microscopy (POM) images (50 × 50 µm) show the average sizes and numbers of spherulites in PBS, PBS/CNC, and PBS/BS‐CNC (**Figure** **3**a‐c and Figure S2a,b, Supporting Information). The interactive 3D graphics clearly reveal the morphologies of the spherulites. In general, the addition of the nano‐filler decreases the average spherulite size and increases the number of spherulites with lower transmittance (Figure S2c, Supporting Information). BS‐CNC was observed to be a more‐effective nucleator than pristine CNC, which is ascribable to its improved dispersibility in the matrix.^[^ ^42^, ^43^ ^]^ Differential scanning calorimetry (DSC) results are provided in the Supporting Information (Figure S3a,b and Table S1, Supporting Information).

The nanocellulose filler dispersion in the PBS matrix was investigated by examining the rheological behavior of each composite in angular‐frequency sweep mode. PBS/BS‐CNC presented the highest complex viscosity (η*) among the samples over the entire frequency range (Figure S4a, Supporting Information), which indicates that well‐dispersed BS‐CNC enhances interfacial adhesion between the filler and the matrix. The homogeneities of PBS and the composites were evaluated from Cole–Cole plots of the logarithm of the storage modulus (G′) versus loss modulus (G″) (Figure S4b, Supporting Information). In general, a homogeneous polymer melt gives a linear plot with two slopes.^[^ ^40^, ^44^, ^45^ ^]^ PBS/BS‐CNC exhibited a Cole–Cole plot that deviates less from those of pristine PBS and the PBS/CNC composite because the modified BS‐CNC surface improves the homogeneity of the composite.

Tensile testing was used to investigate the mechanical‐reinforcing ability of CNC (Figure S4c, Supporting Information). Among the samples, PBS/BS‐CNC exhibited the highest tensile strength of 40.96 MPa, which is 1.12‐times higher than that of pristine PBS, and the formed cracks propagated to the spherulite periphery when fractured. The BS‐CNC composite has a somewhat smaller boundary length than that of pristine PBS (Figure 3a); consequently, cracks struggled to propagate in the BS‐CNC composite in which small spherulites are densely present (Figure 3g,h). Extending the PBS/BS‐CNC spherulite boundary by increasing nucleation density is known to prevent deep crack propagation in the matrix.^[^ ^46^, ^47^ ^]^ In addition, the well‐dispersed BS‐CNC serves to anchor the paper to the polymer matrix because both materials have very similar chemical structures. Interfacial adhesion between the PBS film and the paper was determined using a T‐peel test setup; the materials were regularly adhered by hot‐pressing at 120 °C (Figure 3d). PBS/BS‐CNC presented a higher interfacial toughness (1.31 N mm^−1^) than PBS/CNC (0.18 N mm^−1^) and PBS (0.13 N mm^−1^), which is ascribable to the highly dispersed CNC (Figure 3e,f).

**Figure 4:**
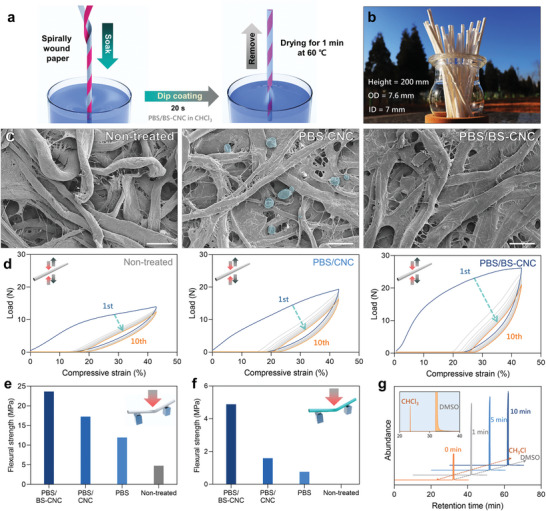
a) Schematic illustrating the paper straw manufacturing step. b) A bundle of PBS/BS‐CNC paper straws and their dimensions. c) FE‐SEM images of the surfaces of non‐treated, PBS/CNC‐, and PBS/BS‐CNC‐coated paper straws. Scale bar: 20 µm. d) Cyclic compression testing of non‐treated, PBS/CNC‐, and PBS/BS‐CNC‐coated paper straws repeated at 10 times. e) Flexural strength comparison for non‐treated, PBS, PBS/CNC, and PBS/BS‐CNC coated paper straw in dry and f) wet conditions (immersed in water for 120 min). g) GC retention times used to analyze residual solvent when dried for various times at 60 °C. Inset: Undried with retention times between 20 and 40 min.

A paper straw was prepared by gluing two pieces of filter paper together using the spiral winding method (**Figure** **4**a). The concentration of the PBS solution was fixed at 5% after evaluating its overall mechanical strength and paper chromatography testing (Figure S5, 6a‐c, and Table S2, Supporting Information). The straw was dipped into a chloroform solution containing PBS or a composite solution containing 5% polymer for 20 s and then dried for 1 min at 60 °C. The composite contained 1% BS–CNCs. The finished product was 200 mm high, with a 7.6 mm outer diameter (OD) and a 7 mm inner diameter (ID) (Figure 4b).

The surface of the paper straw was examined by field‐emission scanning electron microscopy (FE‐SEM, Figure 4c). The PBS/CNC paper showed agglomerated CNC on the surfaces of the paper fibers (blue). On the other hand, the PBS/BS‐CNC paper was relatively smooth compared to the PBS/CNC paper and exhibited no CNC agglomeration. The PBS/BS‐CNC composite coating clearly provides a more homogeneous and smoother surface.

The water repellency of each paper straw was evaluated by determining the water contact angle (Figure S7a‐c, Supporting Information). The uncoated paper immediately absorbed water, while the other coated paper straws exhibited similar contact angles of 82.5°. The addition of CNC does not appear to affect the hydrophobicity of PBS, which explains the observed small difference in contact angle. The contact angle gradually decreased to 67.4° through saturation.

Straws are often soaked in various oily beverages, including coffee and milk. Glycerol contact angles were also measured to evaluate the oil resistance of each paper straw (Figure S8a‐c, Supporting Information). The glycerol contact angle of the non‐coated paper rapidly declined from 73.5° to 25.7°, while the PBS/BS‐CNC‐coated paper resisted glycerol permeation. Clearly, the PBS/BS‐CNC‐coated paper exhibits both water and lipid resistance.

People habitually chew straws with their teeth, and torn paper straws generally provide an unpleasant feel. To simulate these situations, we subjected the paper straws to cyclic compressive testing, the results of which are shown in Figure 4d. The PBS/BS‐CNC paper straw was stronger (26.17 N) than the PBS/CNC and non‐coated straws over 10 testing cycles, and showed the smallest hysteresis (drop to 20.93 N).

Paper straws are easily folded and delaminated under wet conditions owing to hydration and swelling. Therefore, three‐point flexural testing was used to measure the bending strengths of the paper straws under both dry and wet conditions (Figure 4e,f). The dry PBS/BS‐CNC‐coated paper straw was flexurally stronger (23.66 MPa) than the PBS/CNC‐ and PBS‐coated paper straws (17.26 and 11.95 MPa, respectively). The wet PBS/BS‐CNC‐coated paper straw exhibited a flexural strength of 4.88 MPa, while the others showed negligible strengths due to hydration. The residual solvent (chloroform) was determined by gas chromatography (GC) after drying at 60 °C (Figure 4g). No significant chloroform peak was observed after drying for 1 min in a convection oven, which indicates that a PBS/BS‐CNC‐coated paper straw dried for 1 min is safe for human use according to the International Council for Harmonisation of Technical Requirements for Pharmaceuticals for Human Use (ICH) guidelines.^[^ ^48^ ^]^

**Figure 5:**
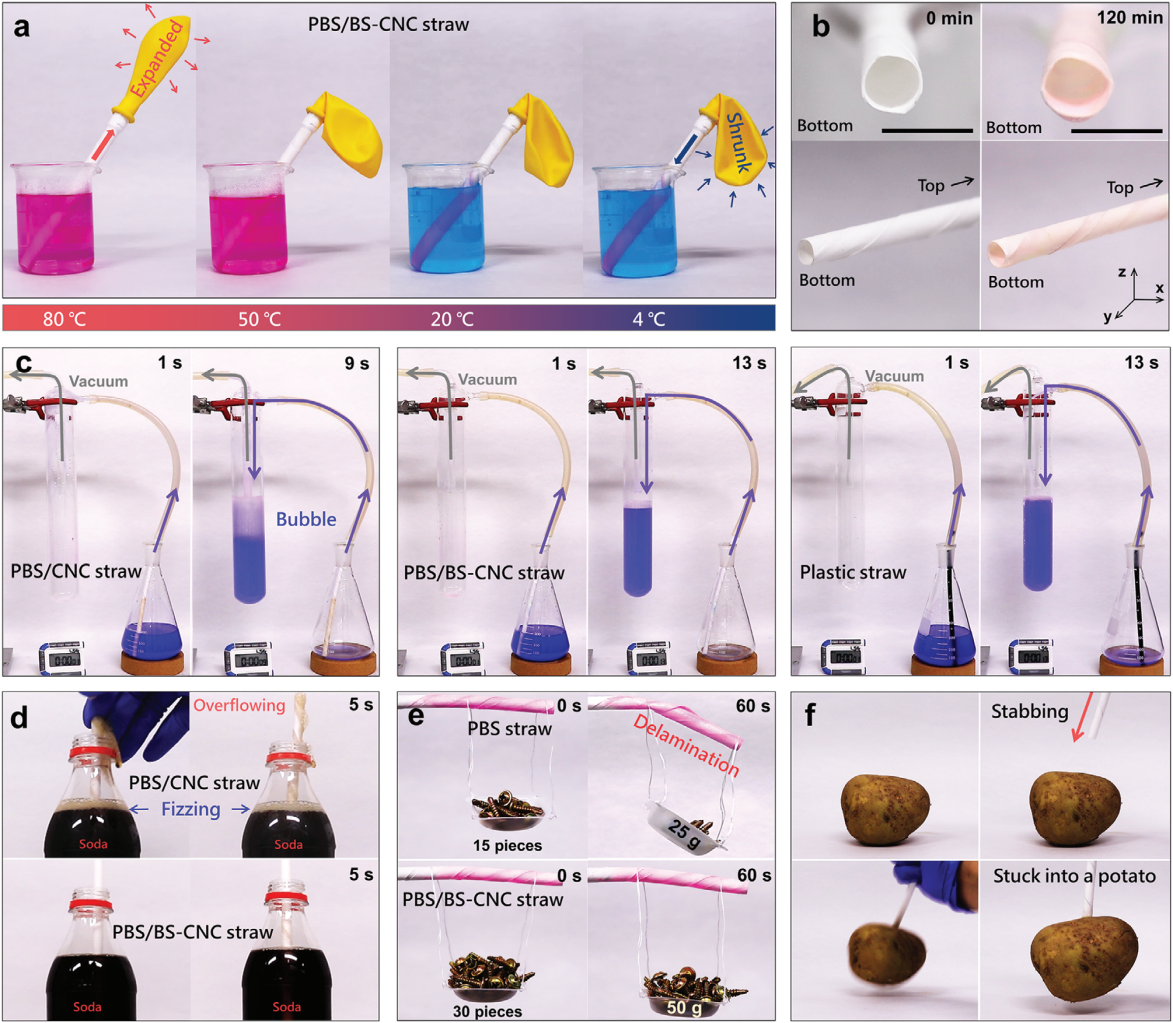
a) Photographic images of PBS/BS‐CNC straws in water in the 4–80 °C temperature range. The upper end of each straw was capped with a balloon for observational purposes. b) Photographic images that show a PBS/BS‐CNC straw before and after immersion in water for 120 min. Scale bar: 10 mm. c) Suction‐testing plastic, PBS/CNC, and PBS/BS‐CNC straws. d) Soda testing PBS/CNC and PBS/BS‐CNC straws. e) Bending PBS and PBS/BS‐CNC straws under wet conditions. f) Stabbing a potato with a PBS/BS‐CNC straw.

People often enjoy drinking beverages that range widely in temperature, including hot coffee, warm tea, and cold soft drinks through plastic straws, and sustainable paper straws are expected to perform as well as traditional plastic straws. PBS/BS‐CNC straws were immersed in water at temperatures in the 4—80 °C range for ≈120 min; these straws were stable without any apparent changes, such as delamination or swelling (as examples), observed, with changes in balloon size confirming that temperature and pressure were applied to the straw (**Figure** **5**a,b).

We simulated drinking a beverage through a straw and measured the amount of suction (pressure) endured by the straw (Figure 5c and Movie S1, Supporting Information). The straw needs to withstand the vacuum created by the user when drinking, otherwise, the straw collapses and becomes blocked; this performance is highly dependent on the rigidity of PBS/BS‐CNC when wet. Water flowed through the straw under the negative pressure of a vacuum pump (16 kPa), which is slightly higher than the inhalation pressure of a human (≈10 kPa).^[^ ^49^ ^]^ The PBS/CNC straw delaminated under these conditions, while the PBS/BS‐CNC straw was able to suck 300 mL of water over 13 s, which is equivalent to that of a plastic straw. The new composite‐coated paper is sufficiently rigid to function as a straw for water and (presumably) other beverages.

The biggest issue that users have with paper straws is that they promote effervescence (fizzing) in soft drinks, which is caused by the highly heterogeneous surfaces of paper straws in terms of uncoated holes and roughness. Supersaturated carbon dioxide evolves at surface defects where uncoated and coated surfaces, or rough and even surfaces attract water and CO_2_ differently.^[^ ^50^ ^]^ Figure 5d and Movie S2 (Supporting Information) show that soft drinks intensively fizz on PBS/CNC and commercial paper straws, while no such bubbling is observed on the PBS/BS‐CNC straw because the well‐dispersed BS‐CNC and its anchoring effect provides a uniform PBS‐matrix coating.

A paper straw often bends slightly or delaminates when used to stir a beverage in a cup (Figure 5e). PBS/BS‐CNC was observed to endure a relatively heavy weight for a long time under wet conditions owing to its high mechanical rigidity in water, which is emphasized by the observation that the straw is sufficiently rigid to stab a potato with (Figure 5f and Movie S3, Supporting Information). As it performs as well as a plastic straw, the PBS/BS‐CNC straw can be used in a variety of beverages, including milk, green tea, soft drinks, and coffee (Figure S9, Supporting Information). Hence, the PBS/BS‐CNC straws are feasible alternatives to traditional plastic straws.

**Figure 6:**
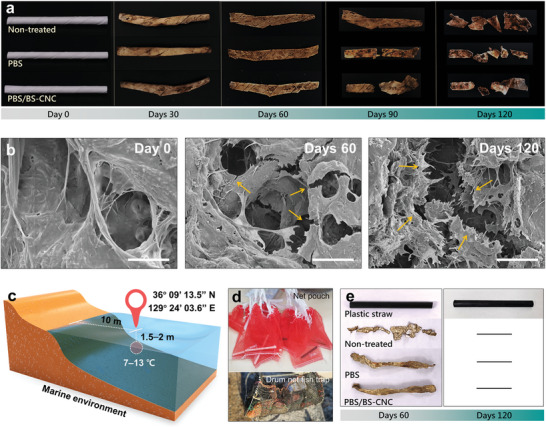
a) Photographic images of straws aerobically composted over 120 d. b) SEM images of aerobically composted PBS/BS‐CNC straws on days 0, 60, and 120. The yellow arrows highlight microcracks. Scale bars: 20 µm. c) Schematic image showing the experimental biodegradability conditions used during marine‐environment testing, along with GPS coordinates. d) Photographic images of biodegradability testing samples. e) Photographic images of straws biodegradability tested in the marine environment for 120 d. The black bars indicate that the samples had disappeared.

Aerobic composting was performed for 120 d to evaluate the biodegradability of the new paper straws (**Figure** **6**a). All paper samples, including the PBS and PBS/BS‐CNC straws, gradually degraded during the composting experiment. Paper is a cellulosic material and PBS is degraded by hydrolysis or enzymatic interactions with aerobic microorganisms.^[^ ^1^, ^51^, ^52^ ^]^ The SEM images in Figure 6b shows that cracked coating layers with tiny holes were observed on the PBS/BS‐CNC straw after 60 d, and significantly more degradation was observed after 120 d.

Biodegradability in the marine environment was also evaluated under static conditions by immersing samples in a net pouch and a drum net fish trap in the sea for 120 d (N 36° 09’ 13.5″, E 129° 24’ 03.6″, Figure 6c,d). Apart from the plastic straws, all samples disappeared during biodegradability testing over 120 d (Figure 6e), which suggests that paper‐based straws are chemically and biologically degraded, even in strongly flowing seawater. As a control, the same marine degradation experiments were performed with a PLA film, which is considered to be environmentally friendly by the public. As a result, there was no noticeable change in its appearance after the 240 d submersion (figure S10, Supporting Information). We confirmed that PBS/BS‐CNC straws can replace plastic straws, which helps to protect the environment.

## 3 Conclusion

In this study, we fabricated biodegradable high‐performance PBS paper straws coated with cellulose nanocrystals surface‐modified with PBS oligomers, which act as eco‐friendly nucleating agents. The PBS/BS‐CNC straw is reasonably mechanically strong under both dry and wet conditions, and exhibits well‐balanced performance compared to commercial products, including thermostability, hydrostability, beverage versatility, compostability, marine biodegradability, and a short manufacturing time. BS‐CNC is regularly dispersed in the PBS matrix, which helps to increase the physical strength of the PBS by reducing its average spherulite size. In addition, its modified groups enhance hydrostability for long‐term applications in a broad range of beverages. Moreover, it appears to overcome defects associated with alternative straws (e.g., oil‐coated paper and PLA straws) and other biopolymers used to manufacture a variety of single‐use products. The PBS/BS‐CNC straws represent a new pioneering bioplastics discovery.

## 4 Experimental Section

Experimental details are provided in the Supporting Information.

## Conflict of Interest

The authors declare no conflict of interest.

## Supporting information

Supporting Information

Supplemental Movie 1

Supplemental Movie 2

Supplemental Movie 3

## Data Availability

The data that support the findings of this study are available from the corresponding author upon reasonable request.
