# Supplementary material for: Biodegradable, Water‐Resistant, Anti‐Fizzing, Polyester Nanocellulose Composite Paper Straws
Source: Adv Sci (Weinh). 2022 Nov 20;10(1):2205554. doi: 10.1002/advs.202205554 (PMC9811439; doi:10.1002/advs.202205554)
Supplement: Supplementary file 1 — Supporting Information [file ADVS-10-2205554-s004.pdf]

ADVANCED SCIENCE
Open Access

Supporting Information

for *Adv. Sci.*, DOI 10.1002/advs.202205554

Biodegradable, Water-Resistant, Anti-Fizzing, Polyester Nanocellulose Composite Paper Straws

Hojung Kwak, Hyeri Kim, Seul-A Park, Minkyung Lee, Min Jang, Sung Bae Park, Sung Yeon Hwang, Hyo Jeong Kim, Hyeonyeol Jeon, Jun Mo Koo, Jeyoung Park* and Dongyeop X. Oh**

Supporting Information

Biodegradable, Water-resistant, Anti-fizzing Paper Straw with Nanocellulose/Polyester Composite

Hojung Kwak, Hyeri Kim, Seul-A Park, Minkyung Lee, Min Jang, Sung Bae Park, Sung Yeon Hwang, Hyo Jeong Kim, Hyeonyeol Jeon, Jun Mo Koo, Jeyoung Park*, Dongyeop X. Oh**

Experimental section

***Materials*:** Succinic anhydride (SA, ≥99%), 4-dimethylaminopyridine (DMAP, ≥99%), pyridine (anhydrous, 99.8%), 1,4-butanediol (BD, ≥99%), titanium(IV) butoxide (97%), rhodamine B, chloroform (anhydrous, ≥99.8%) were purchased from Sigma-Aldrich (Saint Louis, MO, USA), Toluidine blue was purchased from Tokyo Chemical Industry (Tokyo, Japan), PBS was obtained from ANKO Bioplastics (BG5000-M, Wonju, Korea), CNC was purchased from CelluForce (Montreal, QC, Canada), and Quantitative filter paper was purchased from Hyundai micro (No.30, diameter = 300 mm, paper grammage = 84 g m^{-2}, Seoul, Korea).

***Surface modification of CNCs*:** The surface modification was carried out in pyridine under nitrogen atmosphere. CNC (10 g) was dispersed in pyridine (200 g), followed by ultrasonic treatment for 10 min. The mass ratios between CNC and SA were 1:2 (w/w). And also, DMAP as a catalyst was added (0.3 g, 2.46 mmol) in pyridine solution. It was vigorously stirred for 4 h at 70 °C. After the reaction, SA-CNC was washed by acetone and water several times to remove unreacted succinic anhydride. SA-CNC (3g) was esterified with adequate amounts of BD (45.06 g, 0.5 mol) into a flat-bottomed glass reactor using a mechanical stirrer and condenser under nitrogen atmosphere. The flat-bottomed glass was heated to 120 °C for the esterification step. Subsequently, titanium(IV) butoxide (0.06 g, 0.18 mmol) was added to the reactor. The reaction mixture was gradually heated from 120 to 180 °C for 2 h at 5 °C min^{-1}. After that, it was cooled for an hour in ambient condition. The final products were

quenched for removing unreacted monomers in the water bath, which was dried in vacuum oven for overnight at 60 °C

***Preparation of paper straw*:** The poor-hydrostable filter paper was selected as the base material of the straws for proof of PBS/BS-CNC coating performance. First, a filter paper was cut by 25 × 1.5 cm, which was stuck with a glue stick overlapping each other by a gap of 0.5 cm. To replicate a commercial spiral wound paper straw, overlapped paper was rolled up by a steel bar (diameter = 0.7 cm) and then stuck in a diagonal direction with the glue stick. Subsequently, it was dipped into 500 ml of PBS/BS-CNC chloroform solution (Chloroform: PBS: BS-CNC = 100: 5: 0.05, w/w) for 20 sec. Drying was carried out in a drying oven for 1 min at 60 °C.

***Applicability evaluation*:** The end of PBS/BS-CNC straw was capped by a balloon. After that the opposite side was soaked into water in a glass beaker at 80 °C and 4 °C. When the water was cooled down, the shape of the balloon was observed with naked eyes.

The vacuum pump (pressure was 16 kPa) was connected with the straw by a vacuum trap flask. When it started working, the water (300 ml) was thoroughly drawn through straws in a cone flask. Consequently, the water was filled with a vacuum trap flask.

After the bottle of soda was slowly opened, the straw was instantly soaked into the soda. It was observed for 30 sec.

A straw was immersed in water for 120 min, after which a plastic basket was placed on the surface of the straw. The screw (1.68 g) was put on the plastic basket one by one.

Cover the end of the straw with thumb, stabbing it deeply on the skin of the potato.

***Biodegradation test*:** The compost was made of a mixture of hay, fallen leaves, and coffee powder with a weight ratio 1:2:4.[1] It was contained in a 100 liters aluminum container at 50 °C and 60% RH. Each sample was buried in the compost to a depth of 20 cm. The image of samples was captured by digital camera (DSC-RX100M4, Sony, Tokyo, Japan) and the

surface morphology was observed by FE-SEM (Sigma-300VP, Carl Zeiss, Oberkochen, Germany).

Samples were contained in net pouch and drum net fish trap after then, it was immersed under 1.5 to 2 m in natural sea for 120 days (GPS coordinates; N 36° 09' 13.5", E 129° 24' 03.6", Address; Haean-ro 1732 beon-gil, Heunghae-eup, Buk-gu, Pohang-si, Gyeongsangbuk-do, Republic of Korea). The average seawater temperatures were 13.2 °C with the range from 10.9 to 18.4 °C for 4 months. PLA samples were also evaluated as same as PBS straws for 240 d. After collecting samples under the sea, it was carefully washed by D.I. water several times.

General characterizations: The transmittance of coating film was measured with a UV-vis spectrophotometer (UV-2600, Shimadzu CO., Kyoto, Japan) from 400 nm to 800 nm. All samples were cut (1 × 1 cm^2) and attached on the quartz cuvette.

^{13}C-NMR spectra were collected using a QNP probe on a Bruker AvanceII instrument (Billerica, MA, USA) at 150 MHz.

FT-IR spectra were acquired on a Nicolet iS50 FT-IR spectrometer (Thermo Fisher Scientific, Waltham, MA, USA) equipped with an attenuated total reflection (ATR) mode on a diamond/ZnSe crystal. The scanning range was between 4000 and 700 cm^{-1} at a resolution of 4 cm^{-1}, and the number of scans was 128.

The zeta potential of CNC samples was measured using a Zetasizer Ultra (Malvern Panalytical Ltd., Malvern, UK). CNC dispersions of 0.25% at 20 °C, and each sample was totally measured at 5 times. The hydrodynamic particle size was measured for 0.025% CNC dispersions using a Zetasizer Ultra at 20 °C (no salt added). Each sample was measured 5 times, and the average particle size distribution was obtained.

The Tyndall effect was confirmed by a laser beam into the CNC suspension. Each sample was concentrated about 1mg ml^{-1}. The intensity of distance between A and B was measured by ImageJ. (National Institutes of Health (NIH), Bethesda, MD, USA)

POM (BX51TF, Olympus, Tokyo, Japan) was employed to observe the morphology of spherulite growth in PBS, PBS/CNC and PBS/BS-CNC in $CHCl_3$. Each sample (5 mg) was placed on the slide glass and melted on a hitting stage (FP82HT, Mettler-Toledo, Greifensee, Switzerland) at 190 °C for 5 min. And then, it was cooled with rate of 50 °C min^{-1} until 20 °C. Spherulite size, counts and interactive 3D surface plot were measured by imageJ.

The crystallization behavior of samples were measured with non-isothermal DSC (Q-2000, TA instruments, New castle, DE, USA). First of all, degradation temperatures were measured by thermogravimetric analysis. (TGA, Pyris 1, Perkin-Elmer, Waltham, MA, USA) And then, the DSC condition was controlled with heating by 150 °C with a rate of 10 °C min^{-1}. It was cooled at 20 °C with a rate of 10 °C min^{-1}. It was repeatedly heated 150 °C with a rate of 10 °C min^{-1}.

The rheological properties were measured by dynamic oscillation testing using a rotational rheometer (MCR 302, Anton Paar, Graz, Austria), with a parallel-plate geometry with a 25 mm diameter adopted during dynamic frequency sweep testing. The plate gap was 1 mm with disc shape and strain level were 10%, respectively. Before measuring rheological properties, samples were fabricated to disc shapes by the hot-pressing at 190 °C.

The contact angle was measured by using a sessile drop method with an analyzer (DSA-25, Krüss, Hamburg, Germany). The volume of drops was 5 μl, and the images of the droplet were taken with a CCD camera.

Paper chromatography was carried out with 6 papers (100 × 15 mm). Each paper was coated with the mass ratio from 0 to 5% of PBS in chloroform. They were arranged in the same line, which was soaked about 1 cm into Rhodamine B and Toluidine blue mixed water overnight, respectively.

The surface morphology of paper was observed by FE-SEM (Sigma-300VP, Carl Zeiss, Oberkochen, Germany). Each sample was cut (5 × 5 mm) and put into the stubs. It was coated with platinum (Pt) at 15 mV for 120 sec in the sputter coater (Q150R, Quorum Technologies

Ltd., Ashford, UK). The energy of the electron beam was 5 kV and vacuum pressure was around 6×10^{-5} Pa.

Mechanical properties were measured by a universal testing machine (UTM, Instron 5943, Norwood, MA, USA). Tensile strength of coated paper was measured at a speed of 10 mm min^{-1} by 1 kN load cell. The dog bone shaped specimens for testing were followed ASTM D638 Type V (63 × 26 × 0.5 mm in dimensions). Compressive cyclic testing was also measured by 1 kN load cell. Each sample was prepared with the same diameters about 7 mm. The test was repeated 10 times with 40% of percent strain.

Interfacial toughness between paper and PBS matrix was calcuated by performing T-peel test according to ASTM F2256. They were adhered by hotpressing at 120 °C for 30 sec. The width of samples were 10 mm. Peel force was measured at a speed of 10 mm min^{-1} by 1 kN load cell.

The height, inner diameter (ID), and outer diameter (OD) for all the paper straws were ≈200 mm, ≈7 mm, and ≈7.6 mm, respectively. When evaluated three-point bending force in drying and wet conditions (immersed in D.I. water for 120 min), the straw was placed and stabilized on two roller supports at a set distance of ≈80 mm. The beam rested above the two roller supports and was subjected to a concentrated load at the sample's center with a constant test speed of 1.2 mm min^{-1} by a 10kN load cell at room temperature. The flexural strength for tubular structure was derived from following equation.[2,3]

$$\sigma_{tube} = \frac{M \cdot \gamma}{I} = \frac{\frac{F \cdot L}{4} \cdot \gamma}{\frac{\pi \cdot [\left(\frac{D}{2}\right)^4 - \left(\frac{d}{2}\right)^4]}{4}}$$

Where M is the maximum bending moment, I is the moment of inertia of cross section, γ is the maximum distance from the center of the tube (equals to d), L is the support span for the bending test, D is the outer diameter of the straw, and d is the inner diameter of the straw.

As a reference standard, 1mg ml^{-1} of chloroform (in DMSO) was prepared. Multiple drying times: 0 (control), 1, 5, and 10 min were applied. Analysis was performed employing a 7890 B gas chromatography coupled with a 5977 B mass spectrometer (Agilent, Santa Clara, CA, USA). A DB-624 column (60 m × 0.32 mm × 0.8 µm, Agilent) was employed. The oven temperature was initially held at 40 °C for 5 min and subsequently increased to 180 °C at a rate of 18 °C min^{-1} with a hold of 3 min. The mass spectrometer was operated in the electron impact mode at 70 eV, with the mass range set from 50 to 200 m z^{-1}. The interface and ion source temperatures were maintained at 200 and 230 °C, respectively. Helium was employed as a carrier gas at flow rate of 2ml min^{-1}. The sample was analyzed in split mode with a 5:1 split ratio. Target analyte of chloroform was confirmed in the library software installed in the analyzing computer.

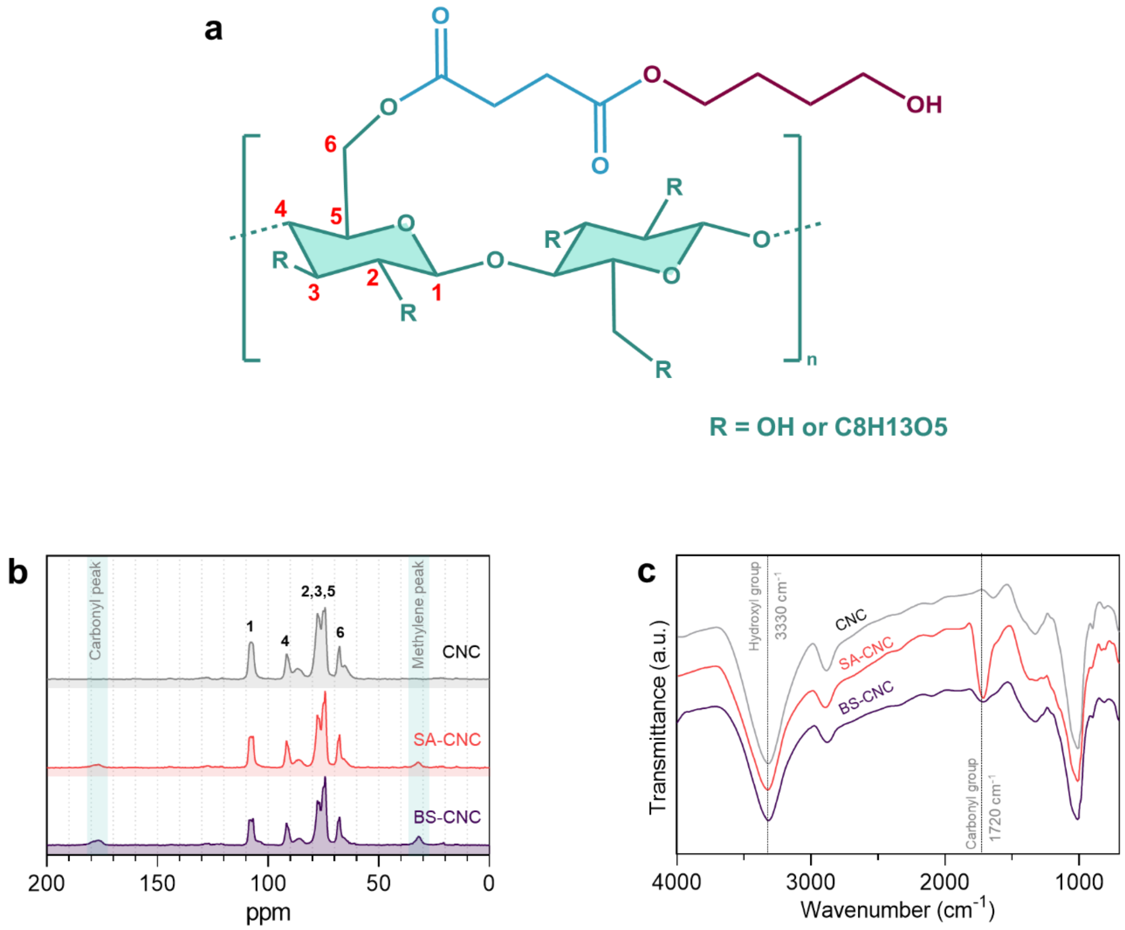

Figure S1. a) The structure of BS-CNC with carbon numbers. b) ^{13}C-NMR spectra of CNC, SA-CNC and BS-CNC. c) The FT-IR spectra of CNC, SA-CNC, and BS-CNC.

The chemical shift of CNC was C6 (68 ppm), C2, 3, 5 (74-77 ppm), C4 amorphous (86 ppm), C4 crystalline (91 ppm) and C1 (107 ppm). SA-CNC and BS-CNC were additionally observed that methylene carbon peak at 32 ppm and carbonyl carbon peak at 176 ppm.

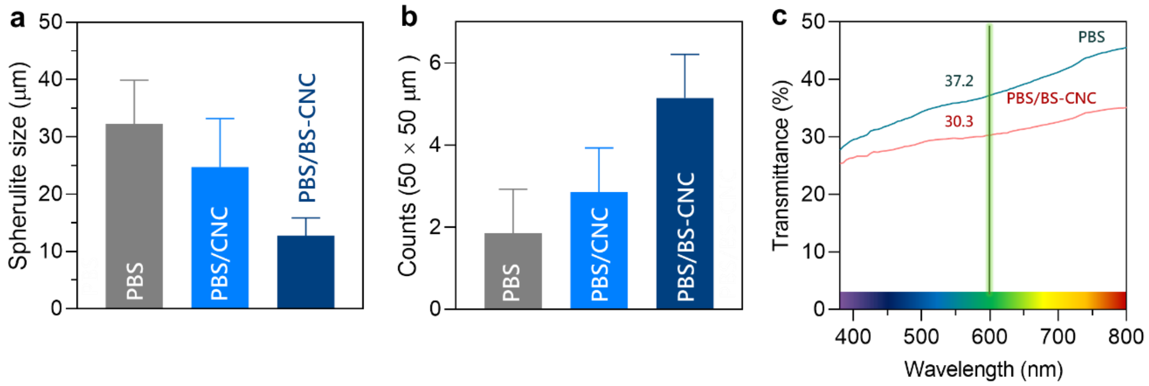

Figure S2. a) The average size of spherulites by random sampling (n = 20). b) The average number of spherulites in a square (50 × 50 μm, n = 7). c) Transmittance of both PBS and PBS/BS-CNC film with the range from 400 to 800 nm. Green dot line was fixed at 600 nm.

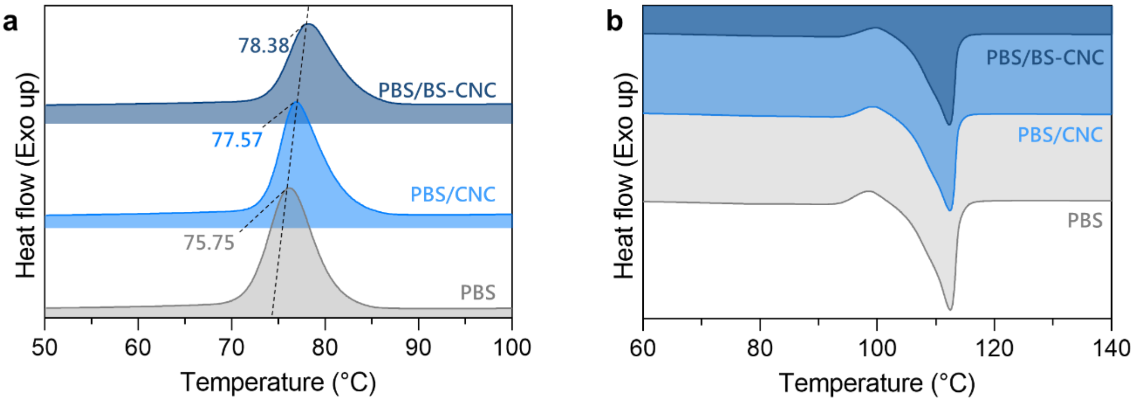

Figure S3. a) Non-isothermal DSC cooling curve indicating T_c with black dash line. b) Second heating curves of PBS, PBS/CNC and PBS/BS-CNC.

Table S1. DSC parameters of PBS, PBS/CNC and PBS/BS-CNC.

	T_c (°C)	ΔH_c (J g^{-1})	T_m (°C)	ΔH_m (J g^{-1})
**PBS**	75.75	56.94	112.52	56.68
**PBS/CNC**	77.57	47.28	112.17	50.68
**PBS/BS-CNC**	78.36	46.85	112.17	46.15

To investigate the crystallization behavior via dispersion state of CNC on the PBS matrix, differential scanning calorimeter (DSC) was measured. As shown in Figure S3a and Table S1 (Supporting Information), the crystallization temperature (T_c) of PBS/BS-CNC was relatively higher than PBS from 75.75 to 78.36 °C on the DSC cooling curves. It was indicated that BS-CNC acted as nucleating agents affected enhancing the rate of crystallization with well dispersed state in the PBS matrix. BS-CNC help them accelerate the formation of nucleation sites by reducing the fold surface free energy. Nucleation density was, in turn, increased as shown in POM images (Figure 3a-3c). There was no notable change in usage with or without BS/CNC on second heating curves (Figure S3b, Supporting Information).

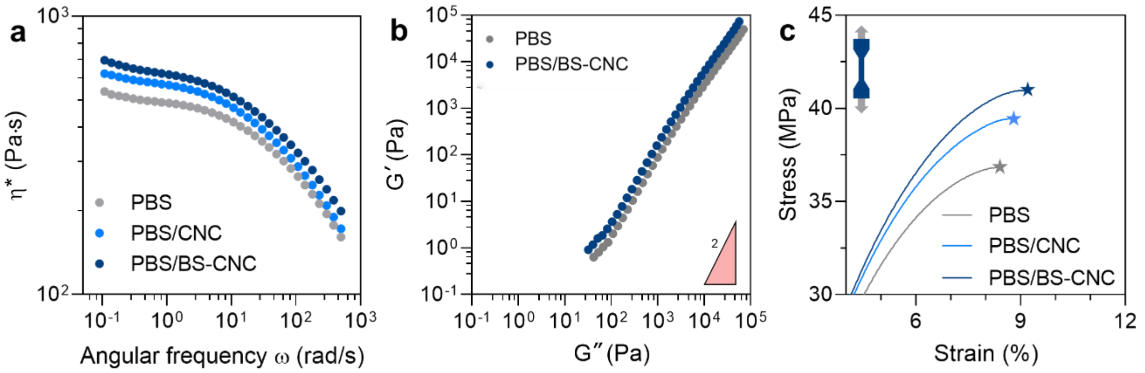

Figure S4. a) The complex viscosity of PBS, PBS/CNC and PBS/BS-CNC. b) The Cole-Cole plot of PBS and PBS/BS-CNC with G′ versus G″. c) Stress-strain curve of PBS, PBS/CNC and PBS/BS-CNC.

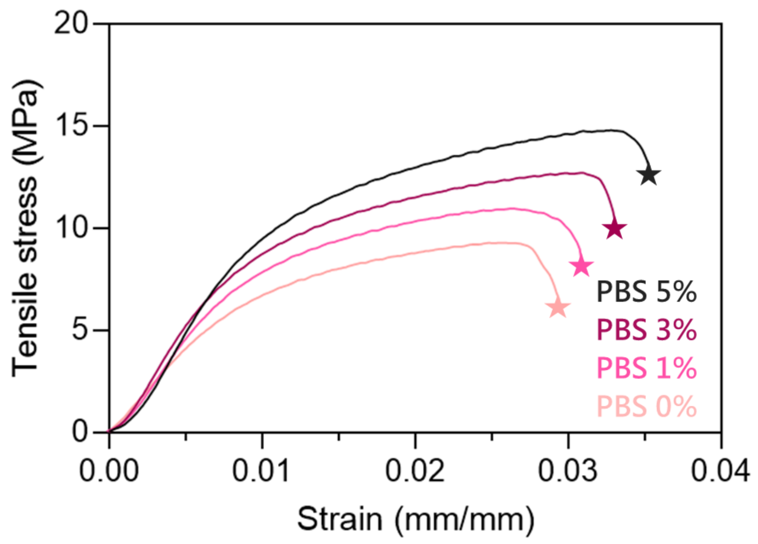

Figure S5. The results of tensile stress of PBS coated filter paper in chloroform.

Table S2. The tensile strength, tensile strain and Young`s modulus of 0,1,3, and 5% of PBS coated filter paper in chloroform.

**Sample**	Tensile strength (MPa)	Tensile strain (%)	Young`s modulus (MPa)
**Control**	8.13	2.81	801.7
**PBS 1%**	11.30	3.12	1045.57
**PBS 3%**	13.50	3.37	1202.85
**PBS 5%**	14.43	3.43	1317.73

Filter paper was coated by dip coating method with concentration from 0 to 5wt% of PBS in chloroform. As a result, tensile strength of PBS coated paper was proportionately enhanced by increasing the concentration of PBS in chloroform (Figure S5 and Table S2, Supporting Information). With this results, the concentration of PBS was fixed at 5% in chloroform. PBS coated paper was soaked into red and blue colored water overnight (Figure S6a-c). Water was instantly absorbed into non coated paper, on the other hand, PBS coating from 3 to 5% helped to resist water absorbing into paper. With these results, the concentration of PBS was fixed at 5%.

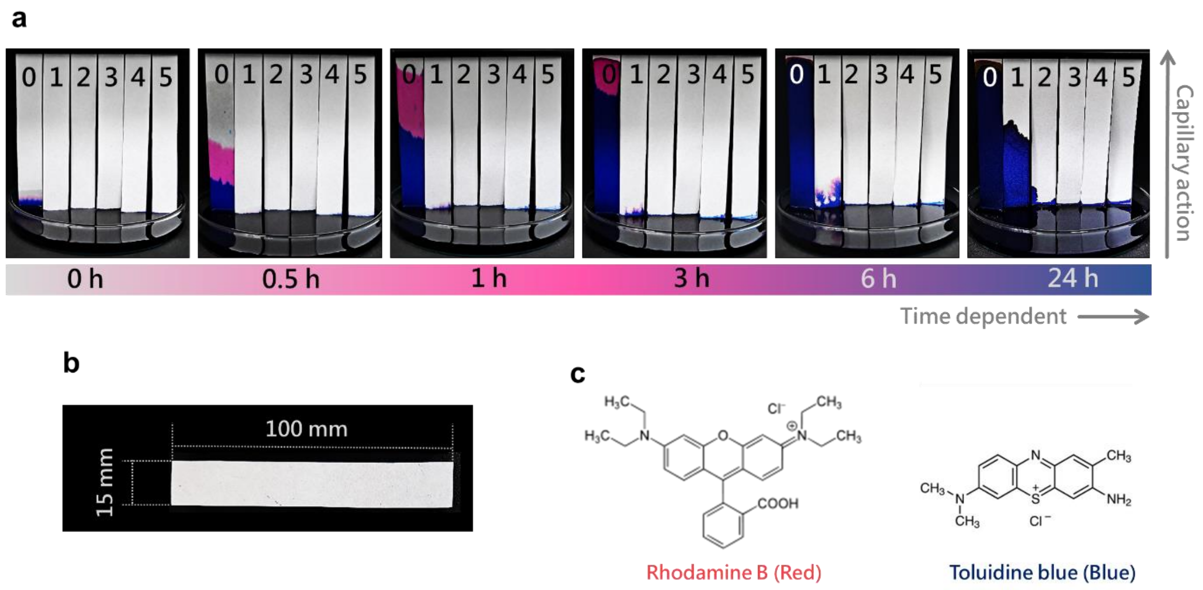

Figure S6. a) Paper chromatography of PBS 0-5% coated filter paper during 24 hours. b) The size of paper chromatography specimen. c) The structure of Rhodamine B and Toluidine blue.

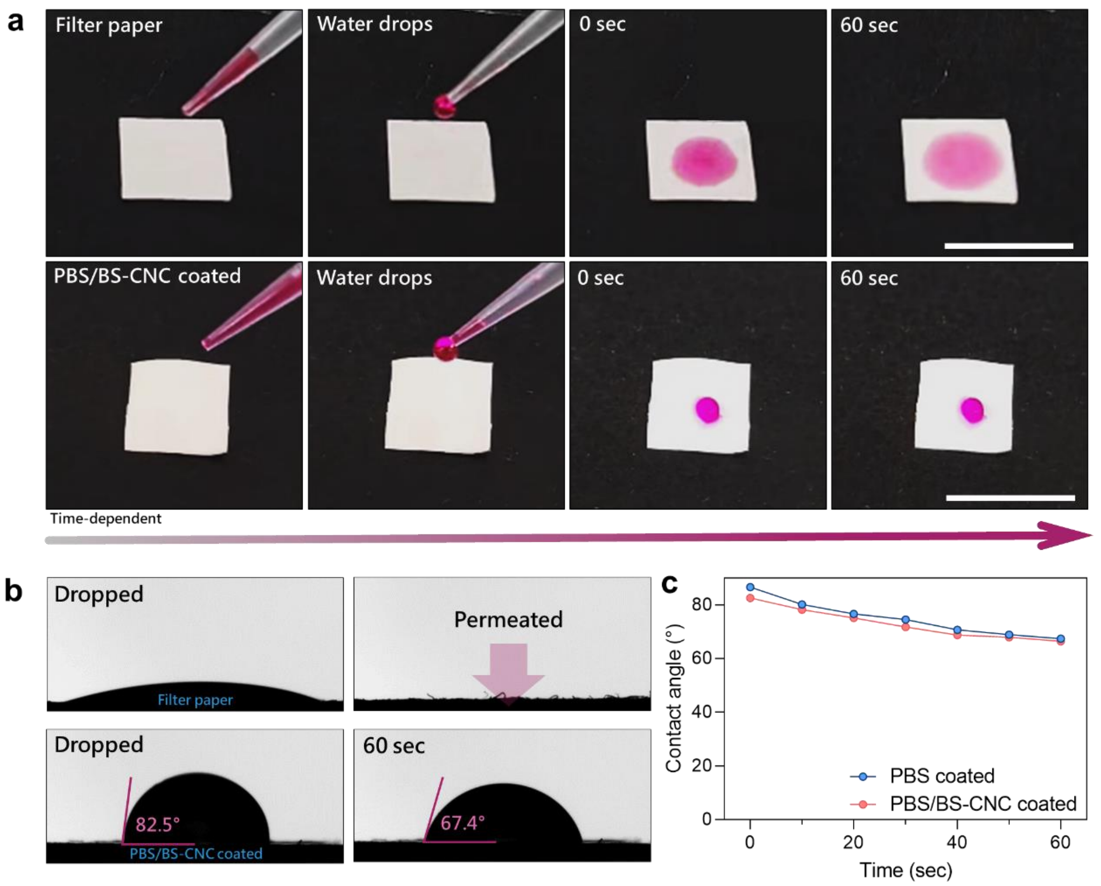

Figure S7. a) The image of water drop on the filter paper and PBS/BS-CNC paper. b) The contact angle of paper by sessile drop method c) The value of contact angle for 60 sec.

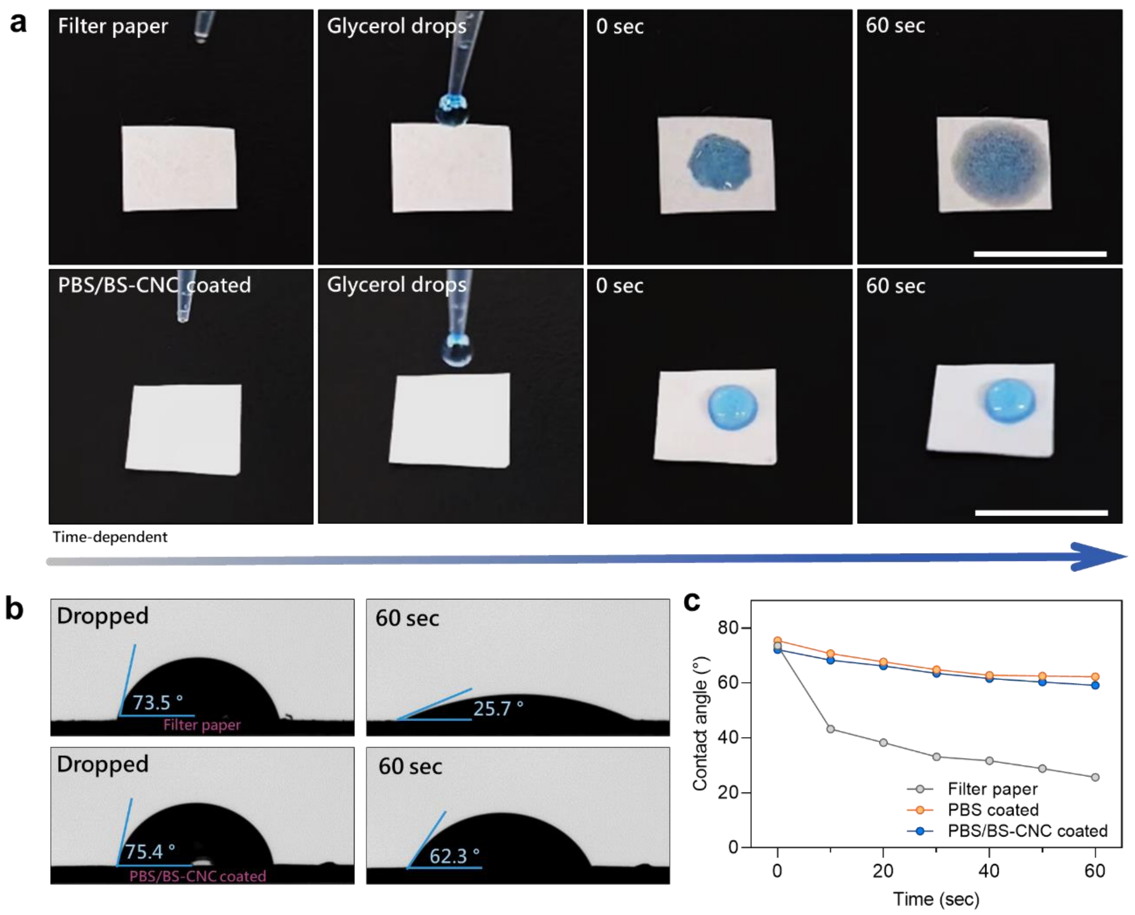

Figure S8. a) The image of glycerol drop on the filter paper and PBS/BS-CNC paper. b) The contact angle of paper by sessile drop method c) The value of contact angle for 60 sec.

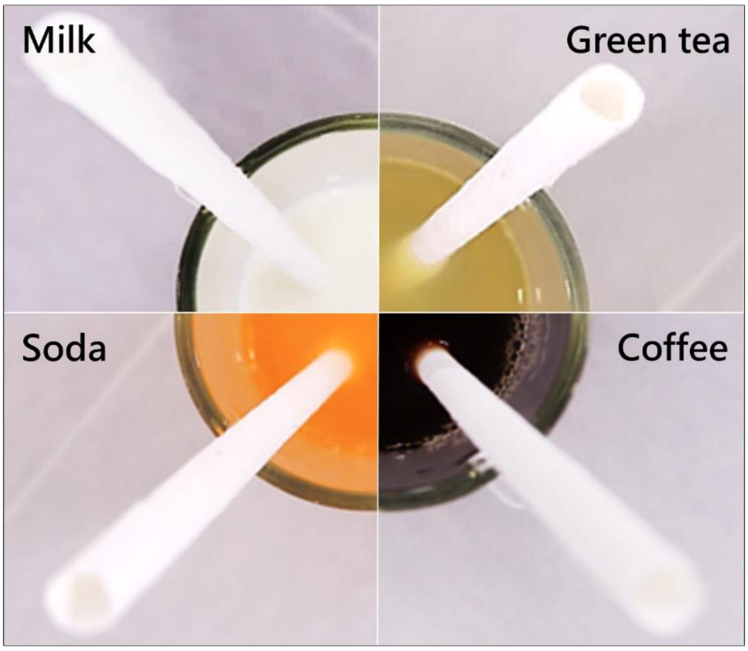

Figure S9. The image of versatility of PBS/BS-CNC straw.

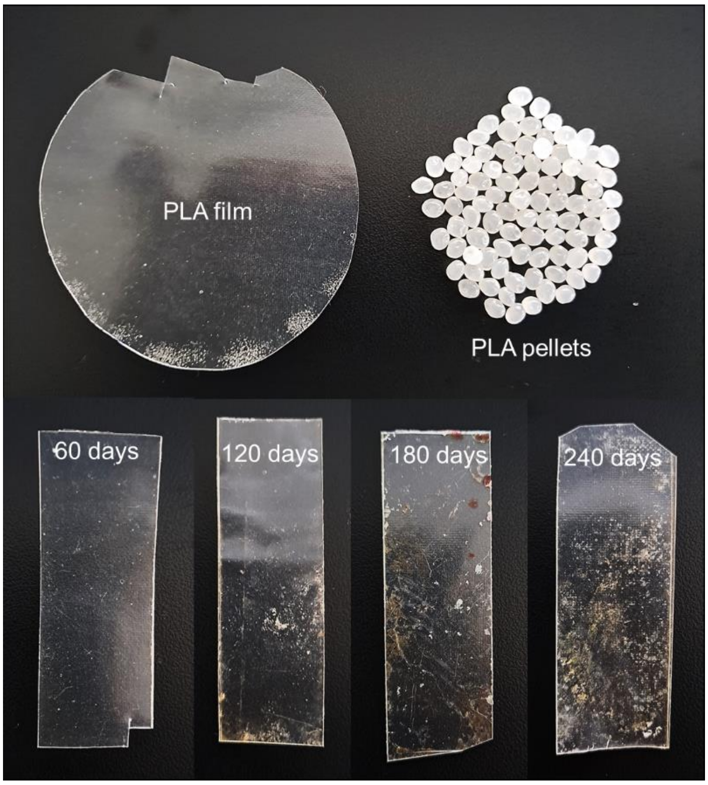

Figure S10. The image of PLA film and pellets with biodegradability test in the marine environment for 240 d.

Biodegradability of PLA in the marine environment was evaluated by immersing samples under static conditions for 240 d (Figure S10, Supporting Information). Unlike PBS/BS-CNC straws, PLA remained in shape for 240 d.

Movie S1. The suction test of plastic straw PBS/CNC and PBS/BS-CNC straw.

Movie S2. Anti-fizzing test of PBS/BS-CNC straw.

Movie S3. The stabbing of a potato with PBS/BS-CNC straw.

References

[1] T. Kim, H. Jeon, J. Jegal, J. H. Kim, H. Yang, J. Park, D. X. Oh, S. Y. Hwang, *RSC Adv.* **2018**, *8*, 15389.

[2] X. Wang, Z. Pang, C. Chen, Q. Xia, Y. Zhou, S. Jing, R. Wang, U. Ray, W. Gan, C. Li, G. Chen, B. Foster, T. Li, L. Hu, *Adv. Funct. Mater.* **2020**, 30, 1910417.

[3] B. Yang, Z. X. Liu, C. H. Yin, Z. M. Han, Q. F. Guan, Y. X. Zhao, Z. C. Ling, H. C. Liu, K. P. Yang, W. B. Sun, S. H. Yu, *Adv. Funct. Mater.* **2021**, 32, 2111713.
